# The CardioMEMS system in the clinical management of end-stage heart failure patients: three case reports

**DOI:** 10.1186/s12872-018-0883-4

**Published:** 2018-07-31

**Authors:** Carsten Tschöpe, Alessio Alogna, Frank Spillmann, Alessandro Faragli, Gunther Schmidt, Florian Blaschke, Uwe Kühl, Ewa Hertel, Monika Willner, Daniel Morris, Heiner Post, Michel Noutsias, Burkert Pieske, Florian Krackhardt

**Affiliations:** 1 Berlin-Brandenburg Center for Regenerative Therapies, Charité – Universitätsmedizin Berlin, corporate member of Freie Universität Berlin, Humboldt-Universität zu Berlin, and Berlin Institute of Health, Campus Virchow Klinikum, Berlin, Germany; 2 DZHK (German Center for Cardiovascular Research), partner site Berlin, Berlin, Germany; 3 Department of Internal Medicine and Cardiology, Charité – Universitätsmedizin Berlin, corporate member of Freie Universität Berlin, Humboldt-Universität zu Berlin, and Berlin Institute of Health, Campus Virchow Klinikum, Berlin, Germany; 4 Department of Cardiology, Contilia Heart and Vessel Centre, St. Marien-Hospital Mülheim, Mülheim, Germany; 5 Mid-German Heart Center, Department of Internal Medicine III, Division of Cardiology, Angiology and Intensive Medical Care, University Hospital Halle, Martin-Luther-University Halle, Halle (Saale), Germany; 6 Department of Cardiology, Deutsches Herzzentrum Berlin (DHZB), Berlin, Germany

**Keywords:** CardioMEMS™, Remote hemodynamic monitoring, Ventricular assist device, Heart failure

## Abstract

**Background:**

Recent clinical trials have shown that pulmonary artery pressure-guided therapy via the CardioMEMS™ system reduces the risk of recurrent hospitalizations in chronic heart failure (HF) patients. The CardioMEMS™ pressure sensor is percutaneously implanted in a branch of the pulmonary artery and allows telemetric pressure monitoring via a receiver. According to the most recent ESC guidelines, this technology has currently a class IIb indication in patients with class III New York Heart Association symptoms and a previous hospitalization for congestive heart failure within the last year, regardless of ejection fraction. Aim of this guided-therapy is multifold, including an early prediction of upcoming decompensation, optimization of patients’ therapy and thereby avoidance of hospital admissions. In addition, it can be used during acute decompensation events as a novel tool to direct intra-hospital therapeutic interventions such as inotropes infusion or left ventricular (LV) assist device monitoring, with the aim of achieving an optimal volume status.

**Case presentation:**

We present a case series of three end-stage HF patients with reduced ejection fraction (HFrEF) who received a CardioMEMS™ device as an aid in their clinical management. The CardioMEMS™ system enabled a closer non-invasive hemodynamic monitoring of these patients and guided the extent of therapeutic interventions. Patients were free from device- or system-related complications. In addition, no pressure-sensor failure was observed. Two patients received a 24-h infusion of the calcium sensitizer levosimendan. One patient showed a refractory acute decompensation and underwent LV assist device (LVAD) implantation as a bridge to cardiac transplantation. Switching a patient with recurrent hospitalizations to the Angiotensin Receptor Neprilysin Inhibitor (ARNI, Sacubitril-Valsartan) on top of the optimal heart failure-therapy improved its subjective condition and hemodynamics, avoiding further hospitalization.

**Conclusions:**

Our case series underlines the potential impact of CardioMEMS™ derived data in the daily clinical management of end-stage HF patients. The new concept to combine CardioMEMS™ in the setting of an outpatient levosimendan program as well as a bridge to LVAD-implantation/heart transplantation looks promising but needs further investigations.

**Electronic supplementary material:**

The online version of this article (10.1186/s12872-018-0883-4) contains supplementary material, which is available to authorized users.

## Background

Heart failure (HF) represents a heterogeneous population of patients defined by multiple etiologies and characteristics sharing, however, a common clinical outcome characterized by disabling symptoms and chronic congestion leading to recurrent hospital admissions [1–3]. The loop of repeated hospitalizations is linked to a high socioeconomic burden estimated to account for around $180 billion only in USA by the year 2030 [4, 5]. Most importantly, within a month of discharge after hospitalization following a decompensation event, the risk of death in heart failure patients clearly peaks [6]. In the past years, different devices have been investigated to help in identifying early decompensation events, modulating the patients’ therapy and subsequently avoiding unnecessary hospital admissions [7–10]. Several interventions had been developed for such purpose, such as study-nurse centered disease management [11, 12] or noninvasive [7, 8] as well as invasive telemonitoring [10, 13, 14]. However, until today only the CardioMEMS™ (St. Jude Medical, Inc., St. Paul, MN) technology has proven reduction in both HF and all-causes related hospitalizations [15, 16]. CardioMEMS™ is an implantable device positioned in the pulmonary artery (PA) able to detect, in patients with HF, higher cardiac filling pressures, an objective measure of “haemodynamic congestion”, estimated to rise more than 2 weeks prior to the onset of symptomatic clinical congestion [17, 18], regardless of left ventricular ejection fraction (LV EF) [19]. The COMPASS-HF (Chronicle Offers Management to Patients with Advanced Signs and Symptoms of Heart Failure) study first demonstrated the safety of this device and allowed a further development of the technology [20]. The CHAMPION trial [21] (CardioMEMS Heart Sensor Allows Monitoring of Pressure to Improve Outcomes in Class III Heart Failure) compared HF hospitalization rates in patients whose therapy was guided by PA pressures (active monitoring group) with patients whose uploaded PA pressures were not available to the clinicians, showing a clear benefit of the pressure-guided therapy. Nevertheless, the role of CardioMEMS™ as a tool to guide intra-hospital therapeutic interventions in end-stage HF patients is still to be investigated. In the real-world scenario several components imbedded best in a heart failure clinic, including educated heart failure nurses, have to be established to identify patients at risk and to develop specific intervention strategies. We here present a case series of three HF patients that received a CardioMEMS™ device as an aid for their clinical management including its potential role for our outpatient intermediate levosimendan program as well as its role for bridge to LVAD-implantation/heart transplantation.

## Case presentation 1

**Fig. 1:**
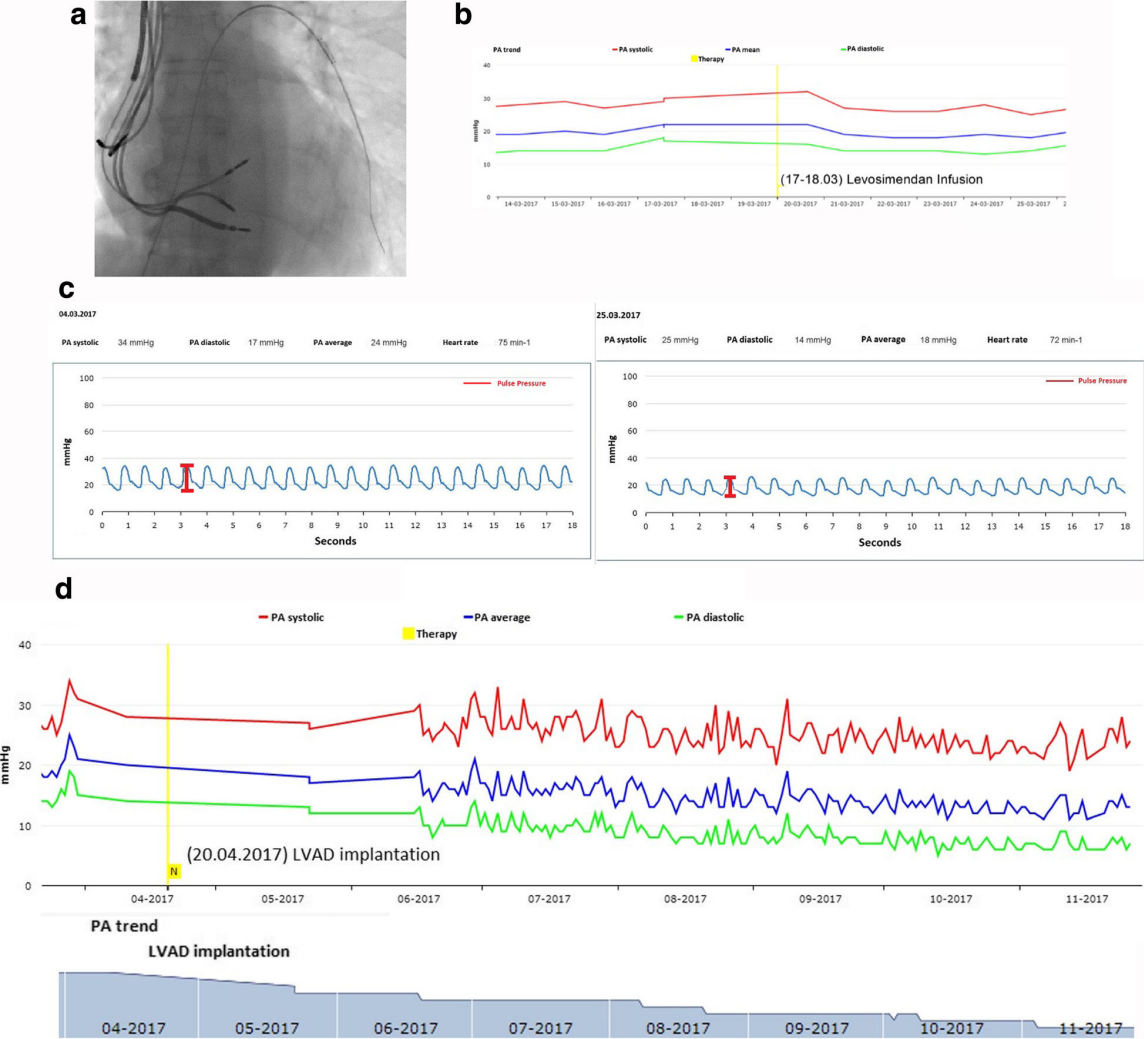
**a** Chest X-Ray showing the positioning of the pressure sensor CardioMEMS™. **b** Pulmonary artery pressure drops after administration of levosimendan. **c** Original single-beat pulmonary artery pressure tracings before and after administration of levosimendan. **d** Pulmonary artery pressure drops over 7 months after implantation of a LV assisted device

A 57-year-old female presented to the clinic with severe dyspnea at mild exertion (NYHA III) and a history of lymphocytic myocarditis. Her comorbidities included stage III chronic kidney disease (CKD), chronic gastritis and Hashimoto thyroiditis. Because of recurring episodes of sustained monomorphic ventricular tachycardia and repeated pre-syncopal events she had received an implantable cardioverter defibrillator in 2009, followed by a cardiac contractility modulation (CCM) – system in 2012. Despite optimal medical treatment (high dose ACEI, ß-Blocker, diuretics and MRA), the patient experienced a severe worsening of dyspnea and quality of life, with a progressive left ventricular ejection fraction (LV EF) reduction and LV dilation during the following years. A coronary heart disease and a recurrence of myocarditis had been excluded by coronary angiography and a repeated endomyocardial biopsy, respectively. For this reason, the patient was enrolled in the waiting list for heart-transplantation and, at the beginning of 2017, a CardioMEMS™ was implanted (Fig. 1a). In the first 3 months, she underwent 2 diuretic dose adjustments. A month later, the CardioMEMS™ documented a rise in pulmonary artery pressure (PAP, 34/24/17 mmHg, Fig. 1b). Therefore she was admitted to the hospital. A transthoracic echocardiogram showed her long-standing dilated cardiomyopathy picture with severe global LV hypokinesia and an ejection fraction of 30%. After excluding any potential cause accounting for the acute presentation, a 24-h infusion of calcium sensitizer levosimendan was administered. At hospital discharge, her basic hemodynamics had improved, as shown by a drop in estimated systemic and pulmonary vascular resistance (1375 and 338 dyn sec cm^− 5^ vs 1167 and 178 dyn sec cm^− 5^ respectively, before and after the infusion). These changes were accompanied by an increased cardiac output (4.5 vs 3.8 l/min). Pulmonary artery mean pressure at 1 week dropped after levosimendan infusion (− 13.5 mmHg x days, calculated as area under the curve change, Fig. 1b), and was correlated with symptomatic improvement. A single-beat view of the PAP before and after levosimendan administration clearly showed a decreased pulmonary mean pressure, as well as a decreased pulmonary pulse pressure at an unchanged heart rate (Fig. 1c). However, despite the initiation of an angiotensin receptor neprilysin inhibitor (ARNI, Sacubitril-Valsartan), which replaced the ACEI, a quick relapse and rise in PAP was observed. Given clinical and hemodynamic worsening despite Levosimendan administration and heart failure therapy optimization, we saw the indication for LVAD-Implantation. A few weeks later the patient underwent a LVAD Heart Mate III implantation as a bridge to heart transplantation. The procedure was uneventful and the patient was discharged home. Since LVAD-implantation, her NYHA class improved to class II, and her hemodynamic parameters have stabilized at lower pulmonary pressures over 7 months (mean PAP constantly below 20 mmHg, Fig. 1d).

## Case presentation 2

**Fig. 2:**
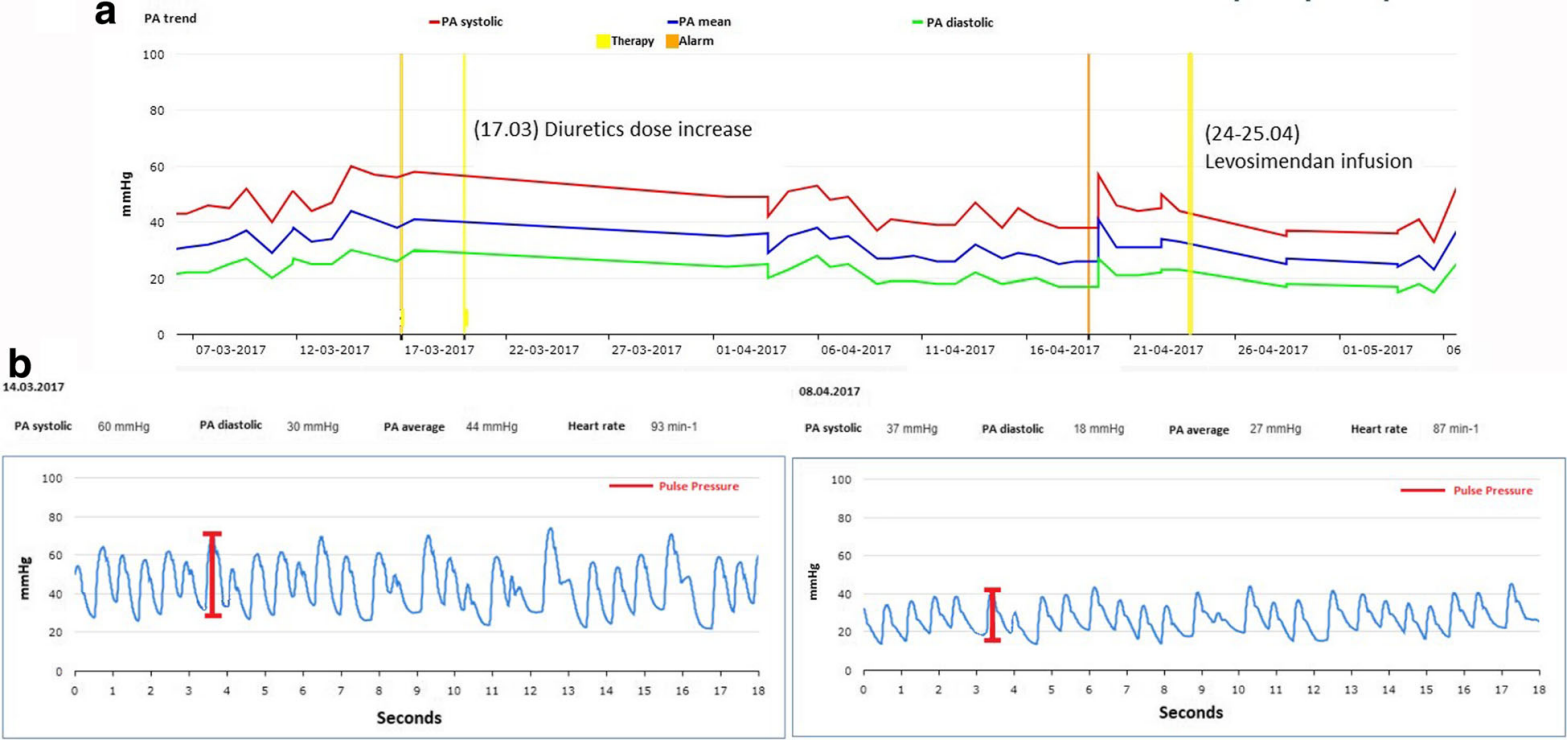
**a** Pulmonary artery pressure tracings showing a mean PAP drop after adjustment of the volemic status (left), as well as after administration of levosimendan (right). **b** Original single-beat pulmonary artery pressure tracings before and after dose-adjustment of diuretics


A 74-year-old male with a history of dilated cardiomyopathy presented to the outpatient clinic with severe dyspnea at rest (NYHA IV). The patient’s comorbidities included arterial hypertension, dyslipidemia, GOLD stage II COPD, stage III CKD, type II-Diabetes, ulcerative colitis and Barrett’s esophagus. His cardiovascular history started in 2008 with recurrent atrial fibrillation episodes and ventricular ectopies of LBBB morphology. He underwent cardioversion and pulmonary vein isolation procedures. A coronary angiography in 2012 revealed a single vessel coronary artery disease, managed conservatively. In 2014 the patient underwent a MitraClip implantation for severe mitral regurgitation. Given the worsening of the patient’s symptoms, recurrent decompensation events, and a severely reduced LV function (LV EF 27%), an implantable cardioverter defibrillator was implanted for primary prevention in June 2015. In February 2016, a baroreceptor simulator was implanted and, given no NYHA class improvement, his medication was implemented with Sacubitril-Valsartan in April 2016. Another decompensation event followed in January 2017 and subsequently a CardioMEMS was implanted. In early 2017, the patient required a diuretic dose adjustment. As shown in Fig. 2a, towards the middle of March 2017, PAP peaked (60/44/30 mmHg), and the patient was suggested to adjust the diuretic dose, allowing an effective reduction in PAP within 3 weeks (37/27/18 mmHg, a single-beat view is shown in Fig. 2b). Given the lack of NYHA class improvement and the sudden PAP rise, a month later the patient was admitted to the hospital for levosimendan infusion. On hospital admission, an echocardiogram was undertaken before inotrope infusion and revealed his previously known dilated LV with severely impaired LV systolic function (EF 27%) and global hypokinesia. After levosimendan administration we observed an improvement in his ejection fraction (LV EF 35%), associated with a mean PAP reduction from a peak of 33 mmHg to 25 mmHg (− 36 mmHg x days, calculated as area under the curve change at 1 week from infusion, Fig. 2a). Two supplemental clips with a 4-chamber-view from the echocardiographic examination before and after levosimendan infusion are available as online supplement (Additional files 1 and 2).

**Additional file 1:** 4-chamber-views from the echocardiographic examination before levosimendan infusion. (MP4 195 kb)



**Additional file 2:** 4-chamber-views from the echocardiographic examination after levosimendan infusion. (MP4 454 kb)


## Case presentation 3

A 53-year-old male presented to our outpatient clinic with severe dyspnea at rest (NYHA IV) and a history of idiopathic dilated cardiomyopathy. His cardiovascular history included the occurrence of paroxysmal atrial fibrillation and ventricular arrhythmias (non-sustained ventricular tachycardia) that were managed with two previous catheter ablations. In 2015, he underwent a coronary angiography as well as left ventricular endomyocardial biopsy sampling that excluded coronary artery disease and myocarditis, respectively. In the same year, a cardioverter defibrillator was implanted (primary prophylaxis of sudden cardiac death). A year ago, he underwent a mitral valve repair with annuloplasty, and percutaneous patent foramen ovale (PFO) closure. Following recurrent hospital admissions with severe decompensation events poorly responded to optimal medical treatment (valsartan 80 mg twice daily, torasemid 5 mg twice daily, bisoprolol 2.5 mg twice daily, eplerenon 25 mg once daily), a CardioMEMS system was implanted in June 2017.

**Fig. 3:**
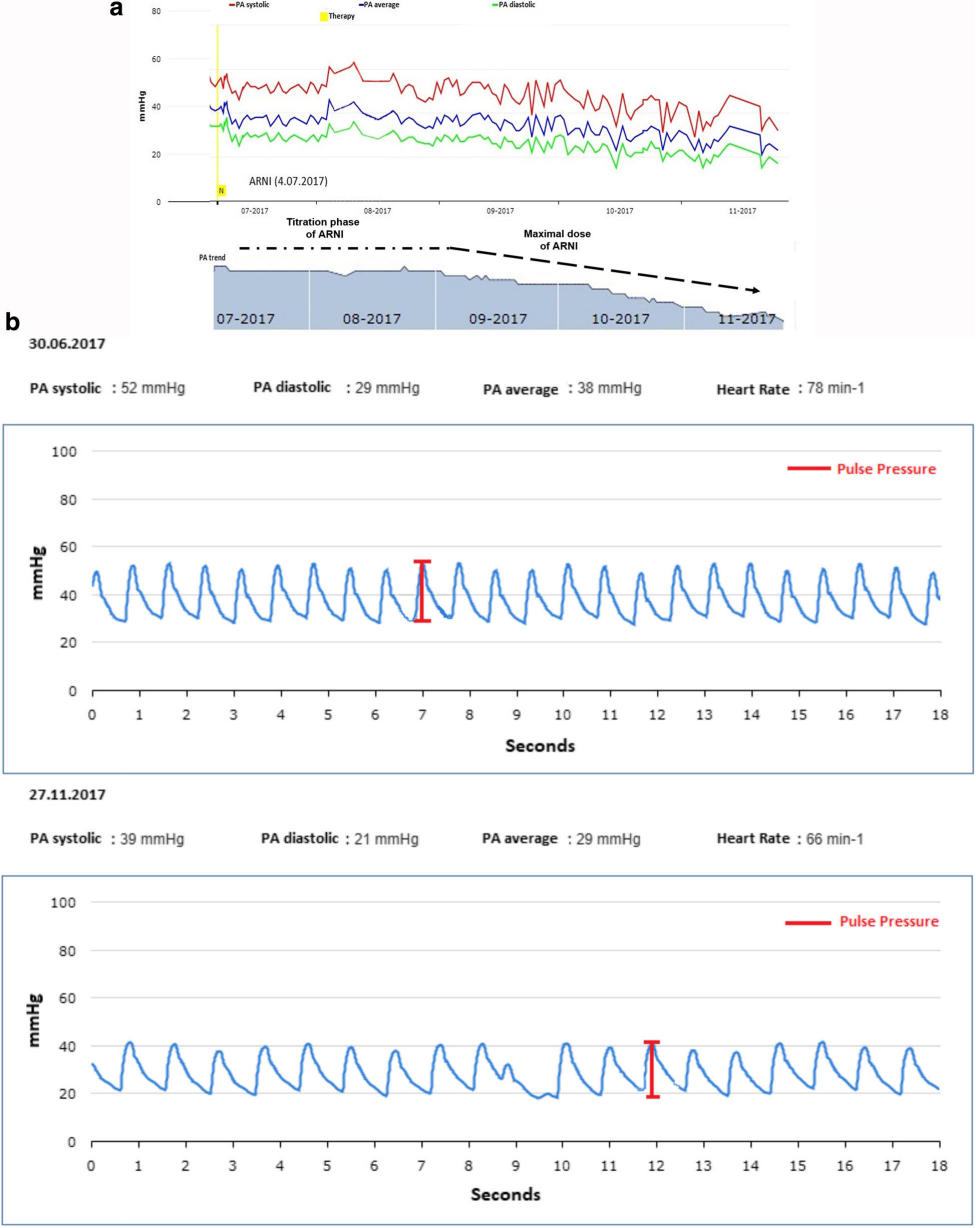
**a** Original pulmonary artery pressure tracings droping after adjustment of the volemic status and switch to the ARNI Sacubitril-Valsartan. Lower panel: Summary of the trend analysing curve indicating the effect of Sacubitril-Valsartan during the titration phase and the effect after reaching maximal tolerated dose. **b** Original single-beat pulmonary artery pressure tracings before and after dose-adjustment of diuretics and switch to Sacubitril-Valsartan

During CardioMEMS implantation a LV end-diastolic pressure of 14 mmHg and a cardiac index of 2.4 l/min were measured. A week post hospital discharge, he had another decompensation event (severe dyspnea and 3 kg weight gain), correlated with a sudden rise in PAP (59/45/35 mmHg) leading to a further hospital readmission. During this hospital stay, his systemic pressure profile and volume status improved on Sacubitril-Valsartan 24/26 mg twice daily and intravenous furosemide 30 mg twice daily respectively, while PAP showed slight improvement (46/33/25 mmHg), (Fig. 3a). The mid-term benefit of switching this patient with recurrent hospitalizations to the ARNI Sacubitril-Valsartan is shown in Fig. 3a. In November 2017, ARNI dose has been increased to 49/51 mg twice daily. Since the first introduction of ARNI, both the patient’s subjective condition, his ejection fraction (LV EF increased from 29 to 39%, LV ESV from 146 to 133 ml, LVEDV from 205 to 219 ml from July to November) and his hemodynamics (a single-beat view from November 2017 is shown in Fig. 3b) have consistently improved, avoiding further hospitalizations. NT-pro BNP decreased from 76,733 ng/l in July 2017 to 1533 ng/l in November 2017.

## Discussion and conclusions

Pulmonary artery pressure-guided therapy is a novel strategy to reduce the risk of recurrent hospitalizations in chronic HF patients, being a class IIb indication in patients with class III New York Heart Association symptoms and a previous hospitalization for congestive HF within the previous year^2^. As most of the literature focuses on the out-of-hospital management of these patients, we set out to discuss the potential of PA pressure monitoring as a guidance to intra-hospital therapeutic interventions in end-stage HF patients. We hereby present a case series of three end-stage HF patients that received a CardioMEMS™ device to improve their clinical management.

In line with the results from the CHAMPION trial [21], the reported patients were free from device- or system-related complications. In addition, no pressure-sensor failure was observed. In our case series, CardioMEMS™ enabled a closer non-invasive intra-hospital hemodynamic monitoring of patients, successfully guiding the extent of therapeutic interventions, while supporting the clinical decision-making. In all three patients diuretic dose was adjusted at least once during the follow-up period, within a median follow-up of 7 ± 2.4 months. This is in line with the data reported in the CHAMPION trial, in which the PA pressure-guided titration of diuretics and vasodilators allowed a 28% reduction in HF hospitalization rates after 6 months compared to the control group [21, 22]. A recent sub analysis from the same trial showed a further reduction in both HF-related and all-cause related 30-day hospital readmissions in patients with high monitoring-compliance [23]. In addition, benefits were sustainable, with a 33% reduction in HF hospital admissions over an average of 18 months of randomized follow-up [24]. Another recent analysis from CHAMPION in the HFrEF cohort demonstrated that the beneficial effects of haemodynamic-guided care were related to a higher level of guideline-directed medical therapies [25], illustrating the synergy between appropriate management of haemodynamic congestion and delivery of guideline-directed medical therapies.

Two out of three patients were admitted to the hospital for a 24-h infusion of levosimendan. A recently discovered novel inotrope, levosimendan, is a calcium-sensitizer shown to combine inotropic, vasodilatory and cardioprotective effects without affecting body oxygen requirements [26–28]. This drug has been shown to be better in comparison with dobutamine in treating HF patients on beta-blocker therapy during acute decompensation [29]. Moreover, it improves symptoms, quality of life and LVEF of both acute and chronic HF patients [30–36], and has currently a class IIb indication [2]. In our clinical series, levosimendan improved hemodynamics and the pressure profiles of our end-stage patients. To the best of our knowledge, no data on levosimendan and CardioMEMS™ monitoring are available in literature.

In the third clinical case, we present for the first-time the telemetric data on the positive impact of the ARNI, sacubitril-valsartan, on our patient’s hemodynamics. The PARADIGM-HF trial [37, 38] compared the effect of this new compound (a combination of the angiotensin II receptor blocker Valsartan with the neprilysin inhibitor sacubitril) to the gold-standard therapy with the ACE-Inhibitor enalapril. This largest clinical trial ever conducted in HFrEF was stopped prematurely due to significant reduction in mortality, demonstrating that potentiation of natriuretic peptide signaling holds great impact for the chronic treatment of HFrEF [37, 38]. In comparison with enalapril, patients treated with ARNI were significantly less likely to have recurrent hospitalizations and were significantly less likely to have one emergency department visit for worsening heart failure. In line with this data, our clinical case suggests that this effect is tightly linked to a better pulmonary pressure profile.

Finally, one of the patients with refractory acute decompensation despite optimal medical therapy underwent LVAD system implantation as a bridge to cardiac transplantation. LVAD patients carry a high readmission rate after implantation, as a result of incomplete LV compensation or pre-existent right ventricular failure. The potential benefit of CardioMEMS™ in managing volume-status and pump performance in such patients still needs to be investigated. Although CardioMEMS™ was helpful in identifying early critical re-decompensation periods of the described HF end-stage patient, already screened for either LVAD or transplantation listing, it was not anymore possible in this clinical scenario to prevent further re-hospitalization or to postpone the need for LVAD. With respect to the costs, it has to be discussed individually whether under these circumstances CardioMEMS™ should be recommended.

Our cases support the universally accepted idea that additional strategies are needed to improve clinical management of end-stage HF patients. CardioMEMS™ was shown to be a safe and effective solution. However, all research to date has been performed within the US healthcare system. Basic differences in HF disease management strategies between countries within Europe as well as between US and Europe might influence the clinical results of haemodynamically guided HF treatment. The ability of HF care facilities in a given health system to provide basic telemonitoring requirements (trained study nurses, patients’ compliance and device training) are essential for the successful management of patients, and might therefore also influence the clinical performance characteristics of the CardioMEMS™ system.

In conclusions, CardioMEMS™ represents a significant technology for reducing the burden of HF in real life scenarios, holding the potential for addressing the urgent need for appropriate HF-management strategies in Germany, as well as in whole Europe [37]. However, further national and international studies/registries are necessary to determine whether clinical benefits of CardioMEMS™ observed in the US are reproducible in a real-world cohort of patients. To address this issue, we have recently initiated a multicentric study, the “prospective CardioMEMS Monitoring Study for Heart Failure (MEMS-HF)” [39]. Aim of the study will be to provide robust evidence on the clinical safety and feasibility of implementing haemodynamic monitoring as a novel disease management tool in routine out-patient care in selected European healthcare systems. The new concept to combine CardioMEMS™ in the setting of an outpatient levosimendan program as well as its role as a bridge to LVAD-implantation/heart transplantation looks promising but needs further investigations.

## Abbreviations

ACEI: Angiotensin converting enzyme inhibitor
Afib: Atrial fibrillation
ARNI: Angiotensin receptor neprilysin inhibitor
CCM: Cardiac contractility modulation
CKD: Chronic kidney disease
COPD: Chronic obstructive pulmonary disease
HF: Heart failure
HFrEF: Heart failure with reduced ejection fraction
LBBB: Left bundle branch block
LV: Left ventricle
LVAD: Left ventricular assist device
LVEF: Left ventricular ejection fraction
MRA: Mineralocorticoid receptor antagonist
NYHA: New York Heart Association
PA: Pulmonary artery
PFO: Patent foramen ovale
